# Enlarged waist combined with elevated triglycerides (hypertriglyceridemic waist phenotype) and HDL-cholesterol in patients with heart failure: Cintura aumentada combinada a triglicerídeos elevados (fenótipo da cintura hipertrigliceridêmica) e HDL-colesterol elevado em pacientes com insuficiência cardíaca

**DOI:** 10.1590/1516-3180.2016.004519102016

**Published:** 2017-01-05

**Authors:** Camila Weschenfelder, Aline Marcadenti, Airton Tetelbom Stein, Catarina Bertaso Andreatta Gottschall

**Affiliations:** I Nutritionist, Instituto de Cardiologia/Fundação Universitária de Cardiologia (IC/FUC), Porto Alegre (RS), Brazil.; II PhD. Professor, Instituto de Cardiologia/Fundação Universitária de Cardiologia (IC/FUC), and Adjunct Professor, Department of Nutrition, Universidade Federal de Ciências da Saúde de Porto Alegre (UFCSPA), Porto Alegre (RS), Brazil.; III PhD. Titular Professor, Department of Public Health, Universidade Federal de Ciências da Saúde de Porto Alegre (UFCSPA), Porto Alegre (RS), Brazil.; IV PhD. Adjunct Professor, Department of Nutrition, Universidade Federal de Ciências da Saúde de Porto Alegre (UFCSPA), Porto Alegre (RS), Brazil.

**Keywords:** Cholesterol, HDL, Heart failure, Hypertriglyceridemia, Waist circumference, Cardiovascular diseases

## Abstract

**CONTEXT AND OBJECTIVE:**

The association of serum triglycerides plus waist circumference seems to be a good marker of cardiovascular risk and has been named the “hypertriglyceridemic waist” phenotype. The aim of our study was to investigate the association between the hypertriglyceridemic waist phenotype and HDL-cholesterol among patients with heart failure.

**DESIGN AND SETTING:**

Cross-sectional study in a tertiary-level hospital in southern Brazil.

**METHODS:**

We included patients with heart failure aged > 40 years. Anthropometric assessment (weight, height, waist and hip circumferences) was performed; body mass index (BMI) and waist-hip ratio were calculated and lipid measurements (serum total cholesterol, LDL-cholesterol, HDL-cholesterol and triglycerides) were collected. In men and women, respectively, waist circumference ≥ 94 cm and ≥ 80 cm, and triglycerides ≥ 150 mg/dl were considered abnormal and were used to identify the hypertriglyceridemic waist phenotype. Analyses of covariance were used to evaluate possible associations between levels of HDL-cholesterol and the hypertriglyceridemic waist phenotype, according to sex.

**RESULTS:**

112 participants were included, of whom 62.5% were men. The mean age was 61.8 ± 12.3 years and the mean ejection fraction was 40.1 ± 14.7%. Men and woman presented mean HDL-cholesterol of 40.5 ± 14.6 and 40.9 ± 12.7 mg/dl, respectively. The prevalence of the hypertriglyceridemic waist phenotype was 25%. There was a significant difference in mean HDL-cholesterol between men with and without the hypertriglyceridemic waist phenotype (32.8 ± 14.2 versus 42.1 ± 13.7 mg/dl respectively; P = 0.04), even after adjustment for age, body mass index, type 2 diabetes mellitus, use of statins and heart failure etiology.

**CONCLUSIONS:**

The hypertriglyceridemic waist phenotype is significantly associated with lower HDL-cholesterol levels in men with heart failure.

## RESUMO

**CONTEXTO E OBJETIVO:**

A associação de triglicerídeos séricos e circunferência da cintura parece ser um bom marcador de risco cardiovascular e é denominada fenótipo da cintura hipertrigliceridêmica. O objetivo do estudo foi avaliar a associação entre o fenótipo da cintura hipertrigliceridêmica e o HDL-colesterol em pacientes portadores de insuficiência cardíaca.

**TIPO DE ESTUDO E LOCAL:**

Estudo transversal em um hospital terciário no sul do Brasil.

**MÉTODOS:**

Foram incluídos indivíduos com insuficiência cardíaca com idade > 40 anos. Foram realizadas as medidas antropométricas (peso, estatura, circunferência da cintura e do quadril) e calculados índice de massa corporal e relação cintura quadril, e foi avaliado o perfil lipídico (colesterol total, LDL-colesterol, HDL-colesterol e triglicerídeos séricos). Em homens e mulheres, respectivamente, circunferência da cintura ≥ 94 cm e ≥ 80 cm e triglicerídeos ≥ 150 mg/dl foram considerados anormais e usados para identificação do fenótipo da cintura hipertrigliceridêmica. Análises de covariância foram usadas para avaliar possíveis associações entre níveis de ­HDL-colesterol e o fenótipo da cintura hipertrigliceridêmica de acordo com o sexo.

**RESULTADOS:**

Foram incluídos 112 participantes e 62,5% eram homens. A média de idade foi de 61,8 ± 12,3 anos e a fração de ejeção média foi 40,1 ± 14,7%. Homens e mulheres apresentaram médias de HDL-colesterol 40,5 ± 14,6 e 40,9 ± 12,7 mg/dl, respectivamente. A prevalência do fenótipo da cintura hipertrigliceridêmica na amostra foi de 25%. Observou-se diferença significativa entre as médias de ­HDL-colesterol entre homens com e sem o fenótipo da cintura hipertrigliceridêmica (32,8 ±14,2 *versus* 42,1 ± 13,7 mg/dl, P = 0,04), mesmo após ajuste para idade, índice de massa corporal, diabetes mellitus tipo 2, uso de estatinas e etiologia da insuficiência cardíaca.

**CONCLUSÕES:**

O fenótipo da cintura hipertrigliceridêmica está associado significativamente com menores níveis de HDL-colesterol em homens com insuficiência cardíaca.

## INTRODUCTION

Heart failure is a complex systemic clinical syndrome^1^ and coronary artery disease is the main cause of heart failure of ischemic origin.^2^

An obesity paradox is commonly reported among patients with heart failure, in which patients with high adiposity have a better prognosis than do individuals who are normal or underweight.^3^ The prognostic value of indexes that detect excess abdominal body fat, such as waist circumference (the traditional tool) and the visceral adiposity index (an alternative and emerging tool) have been evaluated among individuals with ischemic heart failure,^4^ since abdominal obesity is also associated with coronary heart disease.

In addition to abdominal obesity, there has been increasing interest in the role of the atherogenic lipid triad, i.e. hyperinsulinemia, elevated apolipoprotein B and small, dense low density lipoprotein (LDL) particles, in the genesis of coronary artery disease.^5^^,^^6^ However, difficulties in obtaining these parameters in routine practice hinder their use in screening for individuals at high cardiovascular risk. The hypertriglyceridemic waist phenotype (enlarged waist and elevated triglycerides, EWET), defined as simultaneous presence of increased waist circumference and elevated triglycerides, seems to more accurately identify individuals who are at risk, compared with isolated measurements of waist circumference or serum triglycerides,^7^ and can be applied in clinical practice. In addition to the strong association of the hypertriglyceridemic waist phenotype with the atherogenic triad,^8^^,^^9^ it is related to increased visceral adipose tissue,^10^ worse cardiometabolic profile (both in the general population^11^^,^^12^^,^^13^ and in individuals who are at risk^14^^,^^15^ or who present cardiovascular disease^16^), higher incidence of coronary artery disease and cardiovascular mortality.^17^

Low high-density lipoprotein-cholesterol (HDL-c) levels are negatively associated with cardiovascular events in individuals with cardiovascular diseases.^18^^,^^19^ Individuals with the hypertriglyceridemic waist phenotype have been found to present decreased HDL-c levels^11^^,^^12^ and smaller HDL particles.^20^ Gomez-Huelgas et al.^12^ showed that subjects without cardiovascular disease but with the hypertriglyceridemic waist phenotype had lower HDL-c levels independently of sex and age. However, the prevalence of the hypertriglyceridemic waist phenotype was higher in men and it was positively associated with age. In a multiethnic population also without cardiovascular disease,^11^ men with the hypertriglyceridemic waist phenotype showed lower HDL-c levels than women, while HDL-c levels were significantly lower in women with hypertriglyceridemic waist than in those without this phenotype.

Lower levels of HDL-c and higher levels of serum triglycerides may lead to a worse prognosis for ischemic heart disease patients.^21^ Moreover, adipokines secreted by visceral adipocytes may negatively contribute towards decreased HDL-c levels in individuals with heart failure.^22^ Although the hypertriglyceridemic waist phenotype has been investigated in populations in which the obesity paradox is common,^23^ it has not yet been evaluated in heart failure patients.

## OBJECTIVE

To evaluate a possible association between HDL-cholesterol and hypertriglyceridemic waist in men and women with heart failure.

## METHODS

We performed a cross-sectional analysis among patients who had previously been diagnosed with heart failure and who were enrolled at the baseline of a cohort study conducted in a public tertiary hospital. Between 2011 and 2012, these patients were consecutively enrolled if they met the following inclusion criteria: history of New York Heart Association class I-IV heart failure defined by cardiologists in accordance with the American College of Cardiology Foundation/American Heart Association (ACCF/AHA) criteria;^24^ age between 40 and 90 years; no history or clinical evidence of severe heart failure comorbidities (coronary artery disease, cerebrovascular disease or severe kidney disease) over the last six months; and residency in the Porto Alegre metropolitan area (southern Brazil). The following were excluded: patients with lower limb amputation, sequelae of stroke, acute coronary syndrome in the last 90 days or valvular heart disease; pregnant women; candidates for myocardial revascularization; patients in the postoperative period of cardiac surgery (myocardial revascularization or heart valve surgery performed less than one year earlier); and individuals with a history of cancer within the last two years.

Dietitians, medical students and nutrition students administered a questionnaire that asked for clinical data (use of medications, history of diseases, hospitalizations, etc.) and sociodemographic data (age, sex, educational attainment and self-reported skin color). A field coordinator (local cardiologist) was responsible for quality control in relation to the interviews. Patients were also asked about alcohol consumption (alcohol abuse was defined as ethanol consumption per day of 30 g or more among men and 15 g or more among women) and smoking habits, in which they were classified as current smokers, ex-smokers or never smokers.

An anthropometric assessment was performed at the first clinical evaluation. Weight and height were measured with the patient wearing lightweight clothing and standing barefoot on a flat surface, in accordance with the method proposed by Lohman.^25^ Weight was measured to the nearest 100 g using a calibrated scale with a capacity of 150 kg (Cauduro, Brazil). Height was measured to the nearest 0.1 cm using a stadiometer with a measuring rod of 205 cm (Sanny, Brazil). Body mass index (BMI) was calculated in accordance with the World Health Organization criteria, using a cutoff point of 30 kg/m^2^ for the diagnosis of obesity.

Waist and hip circumferences were measured in cm, using an inelastic measuring tape. Waist circumference was measured at the midpoint between the lowest rib and the upper border of iliac crest,^26^ and hip circumference was measured at the maximum protuberance of the buttocks. The waist-hip ratio was calculated by dividing the waist circumference by the hip circumference, and an elevated waist-hip ratio was defined as > 0.90 for men and > 0.85 for women.^27^

The ejection fraction (%) was determined during a transthoracic echocardiogram, using color Doppler and tissue Doppler imaging (GE VIVID 3, General Electric, Norway).^2^ These data were obtained from patients’ medical records. Heart failure etiology was diagnosed by the cardiology staff and was registered in the medical records: ischemic etiology was defined if the individual had a previous diagnosis of ischemic heart disease.

For lipid measurements (serum total cholesterol, LDL-cholesterol, HDL-c and triglycerides), 10 ml of venous blood was collected from each participant. Lipid concentrations were determined using a standard colorimetric enzymatic method. HDL-c levels (dependent variable) were treated as continuous values for statistical analysis. The lipid profile was considered to be altered if the HDL-c level was below 40 mg/dl in men and 50 mg/dl in women, and if serum triglycerides were above 150 mg/dl in men and women,^28^ in addition to the medical diagnosis.

Patients were deemed to present hypertriglyceridemic waist (main independent variable) if they had waist circumference ≥ 94 cm (men) or ≥ 80 cm (women) + serum triglycerides ≥ 150 mg/dl.^28^^,^^29^ Thus, these patients were considered were considered to present the hypertriglyceridemic waist phenotype. Blood pressure was determined using standard techniques, and patients were considered hypertensive if they had previously been diagnosed with hypertension (collected from the medical records), if they had systolic blood pressure ≥ 140 mmHg and/or diastolic blood pressure ≥ 90 mmHg, or if they were taking antihypertensive drugs.^23^ Fasting blood glucose ≥ 126 mg/dl or glycated hemoglobin ≥ 6.5% or a previous medical diagnosis were used to detect patients with type 2 diabetes mellitus.^30^

Sample size was calculated using the WinPepi software, version 11.18. The total sample size required for the study was calculated as 76 individuals, by making the assumptions that the prevalence of hypertriglyceridemic waist phenotype would be at least 20% in the sample, with a difference of at least 7 mg/dl in HDL-c levels between patients with and without the hypertriglyceridemic waist phenotype (standard deviations of 12.3 and 9.4 mg/dl, respectively),^13^ a power of 80% and a significance level of 5%.

Analyses were performed using the Statistical Package for the Social Sciences (SPSS) software, version 17.0 (SPSS, IL, USA). Continuous variables were expressed as means and standard deviations and categorical variables as absolute values and percentages. Student’s t test (continuous variables) and Pearson’s chi-square or Fisher’s exact test (categorical variables) were used for comparisons. Analyses of covariance (ANCOVA) were used to evaluate possible associations between mean HDL-c and hypertriglyceridemic waist after adjustment for potential confounding factors (age, BMI, diagnoses of type 2 diabetes mellitus, statin use and heart failure etiology), separately according to gender. For each analysis, an α-level = 0.05 was considered significant, and 95% confidence intervals (CI) were shown.

The study was approved by the local Research Ethics Committee (CEP-GHC number 10-118), and all patients signed an informed consent statement. There was no external funding for the study.

## RESULTS

Between July 2011 and January 2012, 112 patients were included, of whom 70 (62.5%) were men. Eighty-five patients (approximately 76%) were classified as New York Heart Association grade III-IV. The patients had a mean age of 61.8 ± 12.3 years, and a mean of 5 ± 3.3 years of educational attainment. Thirteen patients (12%) were smokers, 55 (49%) ex-smokers, and 44 (39%) never smoked; 10 patients (9%) were identified as alcohol abusers. Thirty-seven patients (33%) were diagnosed with type 2 diabetes mellitus, 86 (77%) had hypertension and 38 (34%) had dyslipidemia. The mean ejection fraction was 40.1 ± 14.7%, and 19 patients (17%) were diagnosed with ischemic heart failure. The prevalence of hypertriglyceridemic waist phenotype was 25% (95% CI: 16.8-35.6).

The mean BMI was 28.4 ± 6.5 kg/m^2^, and 36 patients (32%) were considered obese (BMI ≥ 30 kg/m^2^). BMI was higher among women (29.7 ± 7.6 kg/m^2^) than among men (27.6 ± 5.8 kg/m^2^), but with no statistical difference. Elevated waist-hip ratio was identified in 91 patients (81%), and the waist-hip ratio values were higher among men (0.99 ± 0.11) than among women (0.93 ± 0.07), but with no statistical difference. Regarding the prevalence of enlarged waist circumference according to different cutoff points for detecting higher cardiovascular risk, for ≥ 102 cm among men and ≥ 88 cm among women, there were 26 cases (23.2%) and 32 cases (28.6%), respectively; for ≥ 94 among men and ≥ 80 among women, there were 37 cases (33%) and 46 cases (41.1%). Triglyceride levels ≥ 150 mg/dl were detected in 32 individuals (28.6%).

No differences between men and women were observed regarding HDL-c levels (40.5 ± 14.6 mg/dl in men and 40.9 ± 12.7 mg/dl in women), systolic arterial pressure (120.1 ± 17.6 mmHg in men and 124.5 ± 18.7 mmHg in women) or diastolic arterial pressure (74.1 ± 11.8 mmHg in men and 75.1 ± 10.9 mmHg in women).

Regarding patients diagnosed with ischemic heart failure, 17 were using statins, of whom three were classified as New York Heart Association grades I and II, and 14 as New York Heart Association grades III and IV, with no statistical difference (P = 0.3) between them. Among the patients with nonischemic heart failure, 36 were using these medications, of whom nine were classified as New York Heart Association grades I and II, and 27 as New York Heart Association grades III and IV, also with no statistical difference (P = 0.9).

Table 1 shows the characteristics of the study group according to presence or absence of the hypertriglyceridemic waist phenotype. Patients with the hypertriglyceridemic waist phenotype had higher prevalence of type 2 diabetes mellitus, dyslipidemia and statin use, higher BMI and ejection fraction and lower HDL-c levels, compared with patients without the hypertriglyceridemic waist phenotype. No statistical difference was observed regarding age, self-reported skin color, educational attainment, smoking, hypertension, New York Heart Association functional classification of heart failure or waist-hip ratio. The prevalence of the hypertriglyceridemic waist phenotype was significantly higher among women than among men (P = 0.01). No patient classified as an alcohol abuser had the hypertriglyceridemic waist phenotype.

**Table 1:**
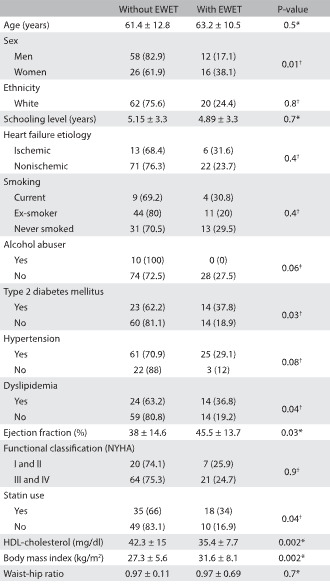
Participants’ characteristics according to presence or absence of hypertriglyceridemic waist (enlarged waist and elevated triglycerides, EWET) [mean ± standard deviation, SD, or n (%)]

*Student’s t test; †Pearson’s chi-square test; ‡Fisher’s exact test. NYHA = New York Heart Association.

Mean HDL-c levels in men and women according to presence or absence of the hypertriglyceridemic waist phenotype are shown in Table 2. In univariate analysis, men with the hypertriglyceridemic waist phenotype had significantly lower (P = 0.001) HDL-c levels than men without the hypertriglyceridemic waist phenotype, but this was not observed among women (P = 0.2). The significant association between the hypertriglyceridemic waist phenotype and HDL-c (P = 0.04) among men was observed even after adjusting for age, BMI, diagnosis of type 2 diabetes mellitus, statin use and heart failure etiology (ischemic/nonischemic) in the multivariate analysis.

**Table 2:**
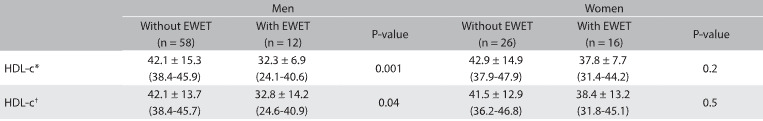
Mean high density lipoprotein-cholesterol (HDL-c) levels in men and women according to presence or absence of hypertriglyceridemic waist (enlarged waist and elevated triglycerides, EWET) [mean ± standard deviation, (95% confidence interval)]

*Univariate analysis, Student’s t test; †Multivariate analysis, using analysis of covariance (ANCOVA) model: mean adjusted for age, body mass index, medical diagnosis of type 2 diabetes mellitus, statin use (yes/no) and heart failure etiology (ischemic/nonischemic).

## DISCUSSION

To our knowledge, this is the first study to evaluate the presence of the hypertriglyceridemic waist phenotype among individuals with heart failure, and also the association of this phenotype with HDL-c levels. We observed high prevalence of the hypertriglyceridemic waist phenotype in the study group (higher among women than among men), which was associated with HDL-c levels in men after adjusting for age, BMI, diagnosis of type 2 diabetes mellitus, statin use and heart failure etiology. Few studies have investigated the hypertriglyceridemic waist phenotype in Brazil; prevalence of 4.5% was reported among young adults^31^ and 33% among Brazilian women with hypertension.^14^

The prevalence of the hypertriglyceridemic waist phenotype varies according to the population studied. Gasevic et al.^11^ compared the prevalence of the hypertriglyceridemic waist phenotype between Aboriginals, Chinese, Europeans and South Asians, and higher prevalence was found among Chinese people, in both genders. The hypertriglyceridemic waist phenotype was reported in 14.5% of the participants in a study conducted in Spain,^12^ and in 41.3% of the individuals with diabetes mellitus in a Serbian population.^14^ The notable differences in prevalence of the hypertriglyceridemic waist phenotype in previous studies may be due to different cutoff points for defining elevated waist circumference in different ethnic groups, and different serum triglyceride values for calculating the hypertriglyceridemic waist phenotype. In the present study, we used the waist circumference and serum triglyceride values proposed in Brazilian guidelines.

Body fat distribution differs between men and women in the general population,^32^ but in our study the frequency of individuals with elevated waist-hip ratio was higher than that of obesity (defined according to BMI), in both genders. Measurement of abdominal adiposity is useful for assessing the risks associated with obesity and excess visceral fat. Visceral adipose tissue, in turn, is metabolically active and associated with insulin resistance, hypertriglyceridemia, small LDL particles and low HDL-c levels.^33^

However, an increased waist-hip ratio may also result from loss of muscle and fat mass from the lower limbs, which is usually associated with the aging process and the pathophysiology of heart failure, particularly the more severe forms. In a study by Fülster et al.^34^ on heart failure patients with a mean age of 66 years, muscle wasting was more pronounced in these individuals than what would be expected for subjects of the same age group. These authors suggested that cachexia relating to chronic heart failure prevails over aging-related loss of lean mass. Therefore, an elevated waist-hip ratio may reflect not only excess abdominal fat accumulation, but also a risk of loss of muscle mass or subcutaneous fat. It is worth mentioning that cardiac cachexia is strongly associated with an inflammatory process.^35^

Hypertrophied visceral adipocytes increase the release of fatty acids via lipolysis and may also contribute towards activation of adipokines involved in inflammation.^36^ As previously mentioned, visceral adiposity plays a role in the pathophysiology of type 2 diabetes mellitus and dyslipidemia. The hypertriglyceridemic waist phenotype can be considered to be an indicator of visceral adiposity that includes anthropometric and biochemical components that are highly associated with a worse cardiometabolic profile and higher prevalence of diabetes, dyslipidemia and statin use. In addition, the higher ejection fraction values observed in patients with the hypertriglyceridemic waist phenotype could be a reflection of the obesity paradox in cases of heart failure, i.e. higher adiposity levels would be associated with lower mortality and hospitalization rates.^3^

In our study, no patients who were identified as alcohol abusers had the hypertriglyceridemic waist phenotype. HDL-c plays a key role in reverse cholesterol transport and attenuates the levels of serum triglycerides. Additionally, ethanol seems to increase HDL apolipoprotein A-I and A-II transport rates by increasing hepatic production.^37^ Therefore, increased HDL-c levels may have contributed towards maintenance of serum triglyceride levels within the normal range (< 150 mg/dl) in the alcohol abusers of our study group. However, we did not evaluate potential associations between other cardiometabolic factors and alcohol consumption.

We found no significant differences in statin use, heart failure functional class and heart failure etiology between patients with and without the hypertriglyceridemic waist phenotype. According to the American Heart Association,^2^ statins should not be used as adjunct therapy in cases of heart failure alone, when no other criteria for their use are met (presence of metabolic syndrome and coronary artery disease). Statin therapy in heart failure patients is controversial, because despite its pleiotropic anti-inflammatory effect, the most effective lipoprotein within the context of cardiovascular risk and protection has not yet been identified.^38^ Higher levels of serum LDL-cholesterol, HDL-c, ApoA-I, ApoB and triglycerides seem to be associated with a better prognosis.^39^

A significant association between the hypertriglyceridemic waist phenotype and HDL-c levels was found among men but not among women, even after adjusting for some confounding variables. This finding may be explained by several factors: first, the markedly higher visceral fat accumulation in men in comparison with women, which is accompanied by elevated serum triglycerides and reduced HDL-c^40^ (although not statistically different, the mean BMI among the women in this study was higher than that of the men, thus suggesting greater subcutaneous fat deposition);^41^ second, the effect of abdominal obesity on proinflammatory states and their atherogenic consequences, including reduction in HDL-c levels;^33^ and finally, changes in HDL-c levels that are commonly observed in heart failure patients, especially those with ischemic heart failure.^22^ The inflammatory process involved in the pathophysiology of heart failure *per se* leads to reduction of HDL-c, which plays a significant anti-inflammatory role in the etiology of the disease. HDL-c inhibits expression of cell adhesion molecules that promote monocyte infiltration through the endothelium, and decreases the inflammatory process that precedes development of heart failure.^42^

Some of the limitations of our study include the facts that this was an exploratory analysis and that the cross-sectional design of the study might point to reverse causality; the small sample size, which may have conferred higher variability and may have lacked power to detect some associations, especially among women; and the fact that the study was carried out in a public tertiary-level hospital that deals with patients with higher prevalence of more severe forms of heart failure, which may limit the generalization of these results.

## CONCLUSION

The prevalence of the hypertriglyceridemic waist phenotype among our patients with heart failure was high. Reduced HDL-c levels were observed in men with the hypertriglyceridemic waist phenotype, even after adjusting for age, general adiposity, statin use and diagnosis of type 2 diabetes mellitus. Further studies are still needed to identify better anthropometric indicators for altered metabolic profiles and better predictors of the risk of cardiovascular events in heart failure patients. Also, further studies on other populations would enable discussion and comparison of our findings.
